# Changes in work conditions and well-being among healthcare professionals in long-term care settings in the Netherlands during the COVID-19 pandemic: a longitudinal study

**DOI:** 10.1186/s12960-023-00847-z

**Published:** 2023-07-28

**Authors:** Renée A. Scheepers, Thijs van den Broek, Jane Murray Cramm, Harry Finkenflügel, Anna Petra Nieboer

**Affiliations:** Department of Socio-Medical Sciences, Erasmus School of Health Policy & Management, Erasmus University Rotterdam, Burgemeester Oudlaan 50, PO Box 1738, 3062 PA Rotterdam, The Netherlands

## Abstract

**Background:**

Healthcare professionals working in long-term care facilities reported heavy job demands and a lack of job resources during the 2019 coronavirus disease (COVID-19) pandemic. However, how job demands and resources in these facilities changed during the pandemic, and how possible changes affected professionals’ work-related well-being, remains unclear. Thus, we explored changes in job demands and resources in the face of surging COVID-19 infection rates, and investigated associations of these changes with changes in burnout and work engagement, among healthcare professionals working in long-term care facilities in the Netherlands.

**Methods:**

This longitudinal study was conducted with healthcare professionals working in five long-term care facilities in the Netherlands. Data were collected in early and late 2021, when infection rates in long-term care facilities were low and high (mean, 29.1 and 275.4 infections/day), respectively. In total, 173 healthcare professionals completed the validated Job Demands and Resources Questionnaire, Copenhagen Burnout Inventory, and Utrecht Work Engagement Scale at both timepoints. We performed paired-samples *t* tests to examine changes in job demands and resources, and fixed-effects linear regression analyses to examine associations of within-person changes in job demands and resources with those in burnout and work engagement.

**Results:**

Healthcare professionals perceived increased workloads, associated with increased burnout and decreased work engagement during the study period. Within-person increases in perceived collegial support were associated positively with work engagement and negatively with burnout symptoms.

**Conclusions:**

Healthcare professionals in long-term care facilities perceived increased workloads in the wake of surging infection rates during the COVID-19 pandemic, resulting in increased burnout and decreased work engagement. These changes in burnout and work engagement were also perceived in response to declining collegial support. Efforts to protect the work-related well-being of healthcare professionals working in long-term care facilities in the pandemic context that focus on workload reduction and the promotion of collegial support may be most beneficial.

**Supplementary Information:**

The online version contains supplementary material available at 10.1186/s12960-023-00847-z.

## Background

Healthcare professionals in long-term care facilities have faced excessive workloads and a heavy emotional burden while caring for frail residents during the 2019 coronavirus disease (COVID-19) pandemic [1, 2]. They have had to continuously adjust work practices and care routines while coping with limited time, resources, and protective equipment [3, 4]. At the same time, they have been burdened with serious concerns about residents’ loneliness due to social isolation and distancing measures implemented during the pandemic [5–7], and have needed to provide intensified psychosocial support to frail residents [3, 4]. Working under these highly stressful circumstances has jeopardized the work-related well-being of these healthcare professionals, who reported burnout symptoms during the COVID-19 pandemic [2, 3, 8].

Burned-out healthcare professionals are emotionally exhausted at work, which undermines their ability to deliver high-quality care [9]. Healthcare professionals working in long-term care facilities have expressed the fear of committing more errors due to exhaustion and burnout during the pandemic [10, 11]. Particularly high burnout levels have been reported by professionals working in environments characterized by heavy job demands and inadequate job resources [8, 12–16]. Job demands are stressful work characteristics that require physical, cognitive, and/or emotional effort (e.g., workloads), and job resources are energizing work characteristics that foster professional growth (e.g., supervisor support). Job demands in long-term care settings were especially high during the COVID-19 pandemic because workloads were heavy and increased exposure to residents’ death and suffering was emotionally burdensome. Job resources were mixed: healthcare professionals in these settings reported strong collegial support, but inadequate support from supervisors [17–19]. However, these findings are drawn from qualitative and cross-sectional studies, and research has not yet provided longitudinal insight into healthcare professionals’ perceptions of actual changes in job demands and resources during the pandemic.

The Job Demands and Resources (JD-R) model postulates two ways in which job demands and resources jointly shape work-related well-being: the *health impairment process* and the *motivational process* [20]. The health impairment process occurs when excessive job demands increase stress and health issues, ultimately resulting in burnout [21]. The motivational process occurs when abundant job resources stimulate professionals’ achievement of work goals and help to reduce job demands, thereby decreasing burnout [20]. It also promotes work-related well-being by stimulating professionals’ work engagement, characterized by energy, dedication, and concentration [22, 23]. Burnout and work engagement have been studied widely and taken to reflect poor and optimal work-related well-being, respectively; burnout is predicted by excessive job demands and insufficient job resources, and work engagement is predicted primarily by abundant job resources [24].

Most research on the predictive value of job demands and resources was conducted before the COVID-19 pandemic [24, 25]. In line with the JD-R model, it has shown that job demands and resources predicting burnout and work engagement vary across contexts and settings [26]. For the long-term care setting, a lack of longitudinal evidence limits insight into the predictive value of job demands and resources for work-related well-being during the COVID-19 pandemic that would allow facilities to optimize conditions to promote such well-being in a pandemic context.

In addition, as the COVID-19 pandemic has involved a continuous flow of infection waves that has forced healthcare professionals to frequently adapt to changing care routines and work practices, marked shifts in job demands and resources likely occurred [4]. Such changes, however, have not been confirmed or characterized, and their associations with changes in healthcare professionals’ burnout or work engagement remain unknown. In the current study, we therefore first addressed the following research question: how did healthcare professionals’ perceptions of job demands and resources change during periods of low and high COVID-19 infection rates in long-term care facilities in 2021? Second, we aimed to answer the research question: how are changes in healthcare professionals’ perceptions of job demands and resources associated with changes in burnout and work engagement?

## Methods

### Study design and sample

**Fig. 1:**
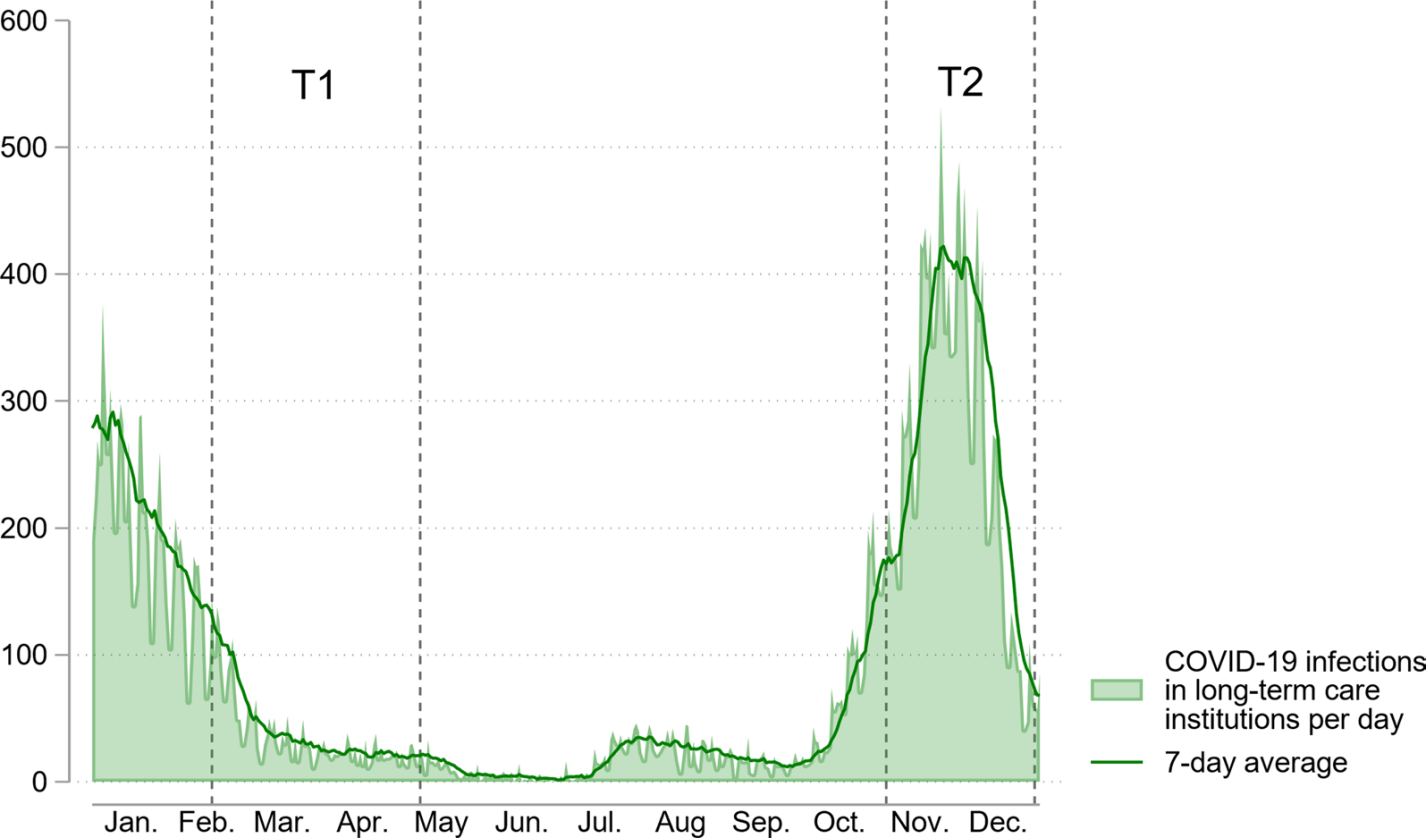
COVID-19 infections per day in Dutch long-term care facilities, 2021. Data are from National Institute for Public Health and the Environment (RIVM, 2022)

Data for this longitudinal study were collected at five long-term care facilities in different regions (southern, southwestern, and central) of the Netherlands in February–May (T1) and November–December (T2) 2021. Infection rates were low [mean, 29.1 (range 11–148) infections/day] at T1 and high [mean, 275.4 (range 41–533) infections/day] at T2 (Fig. 1) [27].

All 6,617 healthcare professionals working at the participating facilities were invited to take part in a web-based baseline survey at T1. They were given approximately 1 month to complete the survey, and (bi)weekly reminders were emailed to increase the response rate. At T2, the same healthcare professionals were invited to complete a follow-up web-based survey.

### Ethical considerations

The Medical Ethics Committee of the Erasmus Medical Center waived the ethical approval requirement for this study (no. MEC-2020-0912). All participants provided informed consent before taking part in the surveys.

### Measures

The web-based surveys at T1 and T2 included validated questionnaires on *job demands*, *job resources*, *burnout* and *work engagement*. *Job demands* (workload, emotional demands, and administrative burden) were measured with the validated Job Demands & Resources Questionnaire (JDR-Q) [28]. Workload was reflected by items addressing the speed and time pressures at work, e.g., “Do you have too much work to do?”. Emotional demands were measured by items addressing the degree to which professionals perceived their work as emotionally burdensome, e.g., “Do you face emotionally charged situations in your work?”. Workload and emotional demands were each assessed by four items, and responses were provided on a 5-point scale ranging from 1 (never) to 5 (very often). Administrative burden—reflecting the degree to which professionals are exposed to administrative tasks and bureaucratic demands—was measured by five items (e.g., “I have to deal with administrative hassles”), with responses provided on a 5-point scale ranging from 1 (totally disagree) to 5 (totally agree). Item scores were averaged per job demand, with higher scores indicating greater workloads, emotional demands, and administrative burdens. All Cronbach’s alpha values for the job demands scales were satisfactory to excellent (workload, 0.89 at T1, 0.93 at T2; emotional demands, 0.80 at T1, 0.77 at T2; administrative burden, 0.87 at T1 and T2).

*Job resources* (collegial support, supervisor support, and autonomy) were also measured with the JDR-Q [28]. Supervisor support addressed the degree to which professionals perceive their supervisors to show appreciation and to support them in solving work-related problems; this was assessed by four items (e.g., “My supervisor shows consideration for my problems and desires regarding my work”). Collegial support and autonomy addressed the degree to which professionals perceive support from their colleagues and experience autonomy in performing their work, respectively. They were each assessed by three items (e.g., “If necessary, can you ask your colleagues for help?” and “Do you have flexibility in the execution of your job?,” respectively). Responses in the three domains were provided on a 5-point scale ranging from 1 (never) to 5 (very often). Item scores were averaged per job resource, with higher scores indicating more supervisor support, collegial support, and autonomy. All Cronbach’s alpha values for the job resources scales were satisfactory to excellent (supervisor support, 0.91 at T1, 0.92 at T2; collegial support, 0.86 at T1, 0.83 at T2; autonomy, 0.78 at T1, 0.77 at T2).

*Burnout* was measured with the work-related burnout and patient-related burnout domains of the validated Copenhagen Burnout Inventory [29]. The domain of work-related burnout reflects exhaustion that professionals attribute to their work in general, and patient-related burnout domain reflects the degree of exhaustion that professionals relate to working with residents. Work-related burnout was measured with seven items (e.g., “Do you feel every working hour is tiring for you?”); four of these items could be completed on a 5-point scale ranging from 1 (never) to 5 (always) and three of these items could be completed on a five-point scale from 1 (to a very low degree) to 5 (to a very high degree). Patient-related burnout was measured with six items (e.g., “Does it drain your energy to work with residents?”); two of these items could be completed on a 5-point scale ranging from 1 (never) to 5 (always) and four of these items could be completed on a five-point scale from 1 (to a very low degree) to 5 (to a very high degree). Item scores were averaged per domain, with higher scores indicating more burnout. Cronbach’s alpha values for the burnout subscales were good (work-related burnout, 0.87 at T1, 0.90 at T2; patient-related burnout, 0.83 at T1, 0.87 at T2).

*Work engagement* was measured with the validated nine-item Utrecht Work Engagement Scale (UWES-9), addressing the degree to which professionals feel dedicated, energetic and enthusiastic in their work (e.g., “I feel happy when I am working intensely”) [30]. Responses were provided on a 7-point scale ranging from 1 (never) to 7 (always). Item scores were averaged, with higher scores indicating more work engagement. Cronbach’s alpha values for the UWES-9 were good (0.86 at T1, 0.87 at T2).

### Approach to missing values

We used multiple imputation with chained equations to deal with missing values [31]. The assumption underlying this approach is that information is missing at random, that is, that any difference between the distributions of missing and observed values can be explained by the variables included in the imputation model. As recommended by Young and Johnson [31], we used a wide data format to allow the imputation to be informed by observed values from both data collection waves. We imputed 20 datasets, and the findings from their substantive analysis were combined into a single set of results following Rubin’s rules [32].

### Statistical analysis

The analysis was performed in two steps. First, we used paired-samples *t* tests to assess whether respondents’ self-reported job demands and resources changed significantly (*p* < 0.05) between T1 and T2. Second, we estimated fixed-effects linear regression models to examine whether within-person changes in job demands and resources were associated with changes in burnout and work engagement. Given that only within-person variance in explanatory and outcome variables was considered, such models account for all time-invariant characteristics (e.g., sex of participants or LTC facility where participants worked during the current study), including those not observed [33]. The models included a dummy variable distinguishing T1 and T2 to avoid the capturing of autonomous temporal trends by the explanatory variable estimates. They were estimated with robust standard errors to account for the nested structure of the data [34]. We also performed robustness checks by re-estimating all models with the non-imputed complete cases subsample. The analyses were performed using Stata 17 [35].

## Results

In total, 876 professionals participated in the T1 survey (response rate, 14.3%); 173 of these respondents completed a follow-up web-based survey at T2 (retention rate, 19.7%). At least one value for a variable of interest at one timepoint was missing for 44 (25.4%) respondents. Table 1 provides an overview of the sample characteristics at T1 after multiple imputation, showing 88.3% female healthcare professionals and average age of 49.7 years.

**Table 1:** Sample characteristics at baseline (*n* = 173); means and percentages

Continuous variable	Mean	(Standard deviation)
Work-related burnout	2.4	(0.6)
Patient-related burnout	2.0	(0.6)
Work engagement	4.7	(1.0)
Age	49.7	(12.3)
Years of experience	16.6	(13.3)

Categorical variable	Percentage
Female	88.3
Job type	
Nursing staff	56.0
(Para)medical staff	18.6
Support staff	25.5
Educational attainment	
Lower secondary or less	15.1
Higher secondary or lower tertiary	56.4
Higher tertiary	28.5

Multiple imputation using chained equations used to deal with missing data

The respondents reported significantly greater workloads at T2 than at T1 (Δmean = 0.122, *p* < 0.01, see Table 2). No significant difference in the other job demands (emotional demands and administrative burden) was observed (Table 2). We also found no evidence that job resources changed between T1 and T2.

**Table 2:** Changes in job demands and job resources (*n* = 173)

	T1 (Feb. –May 2021)		T2 (Nov.–Dec. 2021)		T2 versus T1	
	Mean	(SD)	Mean	(SD)	ΔMean	(SE)
Job demands						
Workload	3.306	(0.738)	3.429	(0.824)	0.122**	(0.046)
Emotional demands	3.080	(0.6451	3.039	(0.595)	− 0.040	(0.040)
Administrative burden	3.087	(0.770)	3.139	(0.772)	0.055	(0.043)
Job resources						
Supervisor support	3.414	(0.799)	3.368	(0.815)	− 0.046	(0.058)
Collegial support	4.027	(0.715)	3.951	(0.677)	− 0.076	(0.054)
Autonomy	3.749	(0.630)	3.762	(0.619)	0.013	(0.047)

Multiple imputation using chained equations used to deal with missing data; SD: standard deviation; SE: standard error;

^**^p < 0.01

The regression model showed that within-person increases in workload (*b* = 0.227, *p* < 0.01) emotional demands (*b* = 0.267, *p* < 0.001), and administrative burden (*b* = 0.223, *p* < 0.01) were associated with significant increases in work-related burnout (Table 3). Increases in collegial support (*b* = − 0.114, *p* < 0.05) and autonomy (*b* = − 0.115, *p* < 0.05) were associated with declines in work-related burnout. No significant association of changes in supervisor support and work-related burnout was observed (Table 3).

**Table 3:** Results of fixed-effects regression analyses predicting work-related well-being changes

	Work-related burnout		Patient-related burnout		Work engagement	
	*b*	(SE)	*b*	(SE)	*b*	(SE)
Job demands						
Workload	0.227**	(0.074)	0.215*	(0.089)	− 0.344*	(0.147)
Emotional demands	0.276***	(0.071)	0.171*	(0.073)	0.060	(0.142)
Administrative burden	0.223**	(0.072)	0.160^†^	(0.091)	− 0.008	(0.140)
Job resources						
Supervisor support	− 0.061	(0.058)	− 0.024	(0.063)	0.038	(0.123)
Collegial support	− 0.114*	(0.054)	− 0.046	(0.069)	0.286*	(0.130)
Autonomy	− 0.115*	(0.054)	− 0.026	(0.071)	0.244^†^	(0.124)
Time period						
T1 (Feb.–May 2021)	Ref		Ref		Ref	
T2 (Nov.–Dec. 2021)	− 0.001	(0.033)	0.020	(0.039)	− 0.008	(0.074)
Number of observations	346		346		346	
Number of persons	173		173		173	

Multiple imputation using chained equations used to deal with missing data; all models estimated with robust standard errors; B: coefficient estimate; SE: standard error

^†^*p* < 0.1, *p < 0.05, **p < 0.01, ***p < 0.00

Increases in workload (*b* = 0.215, *p* < 0.05) and emotional demands (*b* = 0.171, *p* < 0.05) were associated with elevated patient-related burnout. We found no significant association between changes in administrative burdens or job resources and changes in patient-related burnout (Table 3). Increases in workload were associated with declines in work engagement (*b* = − 0.344, *p* < 0.05) and increases in collegial support were associated with increased work engagement (*b* = 0.286, *p* < 0.05). No significant association of a change in work engagement with other job demand or resource was observed (Table 3).

The analysis of the complete cases subsample yielded results consistent with those of the main analysis. Specifically, respondents’ workloads increased from T1 to T2 (ΔMean = 0.149, *p* < 0.01, Additional file 1: Table SA) and seven of nine associations that were significant in the main analysis were also significant in the subsample (Additional file 1: Table SB). The remaining two associations (of autonomy with work-related burnout and workload with patient-related burnout) were similar in direction and magnitude, but only marginally significant (*p* < 0.1). Some estimated effects were also significant in the subsample. Specifically, the effect of administrative burden increased significantly and that of collegial support decreased significantly between T1 and T2. Moreover, the association between changes in autonomy and those in work engagement was similar in direction and magnitude to that observed in the main analysis, but significant in the subsample.

## Discussion

### Main findings

Our analyses showed that healthcare professionals in long-term care facilities in the Netherlands experienced notably greater workloads during the COVID-19 the pandemic when the infection rate was higher than when it was lower. These increased workloads were associated with elevated work-related and patient-related burnout and declines in work engagement. Furthermore, within-person increases in collegial support were associated positively with work engagement and negatively with work-related.

### Interpretation of findings

The findings of this study contribute to the body of knowledge about healthcare professionals’ perceptions of job demands and resources during the COVID-19 pandemic, which to date has largely been cross-sectional and qualitative [2, 18, 19], by providing longitudinal insight against the backdrop of surging infection rates. In this period during the pandemic, we found that workloads increased. Workload during the pandemic may already have been higher than before the pandemic, as earlier research showed that healthcare professionals experienced higher workloads in terms of quantitative demands during the pandemic than reported by a reference group of healthcare professionals before the pandemic [36]. Furthermore, qualitative findings have also shown professionals to perceive excessively high workloads during the pandemic, as fewer staff members had to provide more intensive care to larger numbers of residents due to infected staff members’ absenteeism [37]. Rather than focusing on the situation during the pandemic as a whole or comparing it with the pre-pandemic situation, we demonstrated that the perceived workloads of healthcare professionals in long-term care settings varied across pandemic stages and that changes in job demands (including workload) and job resources were associated with changes in work-related well-being.

The reported increase in workload when the infection rate was higher in this study may be related to the need to adhere to more extensive preventive procedures (i.e., regularly changing personal protective equipment, such as masks and hazmat suits) [36]. These time-consuming procedures were also applicable during the study period and have been suggested to evoke healthcare professionals’ experience of the “technicisation” of care with the limiting of time to provide psychosocial and emotional care. This situation may also partially explain the lack of a perceived increase in emotional demands at T2 among our respondents, as it may have limited healthcare professionals’ ability to devote attention and time to the possibly greater emotional demands of infected and isolated residents.

Professionals in our study who did experience increased emotional demands and workloads when infection rates were high reported increased levels of work-related and patient-related burnout. These findings are consistent with those of cross-sectional pre- and peri-pandemic studies showing that greater workloads and emotional demands are associated with burnout [13, 38–40]. We also found that respondents’ work engagement was affected by changing job demands (workload) and resources (collegial support). The relevance of collegial support to work engagement has been demonstrated in pre-pandemic research [25, 39]. The observed association between workload and work engagement, however, stands in contrast to previous findings. In a pre-pandemic meta-analysis, job demands (including workload) did not predict work engagement [24]. This finding is consistent with the JD-R model, which posits that job demands lead primarily to health impairment and ultimately burnout, whereas job resources trigger the motivational process preceding work engagement [41]. Although work-related health impairment and motivation were proposed as independent processes, researchers have suggested that they be studied jointly, as they reflect two sides of the same coin: when work-related health is impaired (resulting in burnout), this may also negatively affect work-related motivation (in terms of work engagement), and vice versa [42].

Our finding that changes in job demands (workloads in particular) and resources were predictive of work engagement and (work-related) burnout in the COVID-19 pandemic context is in line with the finding of a pre-pandemic longitudinal study conducted outside of the healthcare work setting that job demands (workload and role ambiguity) had cross-lagged impacts on work engagement [43]. A longitudinal study conducted with nurses also showed that work engagement declined in response to the reduction of job resources in a high-demand work environment [21]. The work environment during the pandemic was typically highly demanding, and healthcare professionals perceived that decreasing job resources (collegial support) compromised their work engagement [21]. Consistent with this reasoning, previous work has shown that concepts related to work engagement, such as job satisfaction, are affected by workload and related demands (e.g., staff shortages) [44–46].

### Limitations

This longitudinal study was unique in that data were collected at two timepoints during the pandemic, characterized by low and high infection rates, respectively, and compared. The infection rates of long-term care facilities in our study were, however, not known. We assume that national trends in infection rates were also reflected in institutional infection rates, yet, we could not verify this assumption. Studying perceptions of job demands of resources among professionals across facilities with varying infection rates, could have provided even more convincing evidence of the impact of surges in infection rates on healthcare professionals’ job demands and job resources, and, ultimately, their well-being. Nonetheless, our findings are in line with those of related research conducted in various countries during the COVID-19 pandemic [1, 2], but their generalizability to countries with other long-term care systems needs to be assessed in future research.

The pandemic complicated the achievement of high response and retention rates in this study, as in similar studies [47, 48], given professionals’ lack of time to complete surveys during this period. Furthermore, there was variation in response (8.1–25.7%) and retention rates (11.8–34.4%) across participating long-term care facilities, which may imply that professionals of specific regions may be under- or over-represented in our sample. Therefore, we cannot exclude the possibility of selection bias, which could also be related to possible healthy worker bias: ill healthcare professionals could have been underrepresented in the sample while healthy professionals may be overrepresented. In that case, we may have overestimated the overall levels of work-related well-being at baseline, which may not be necessarily problematic given our study’s focus on within-person changes in work-related well-being, rather than on absolute well-being levels. Furthermore, the possibility of healthy worker bias could also imply that respondents who scored relatively favorable at T1 (e.g., high well-being and job resources, low job demands), but unfavorable at T2 (e.g., substantial declines in well-being or job resources, versus inclines in job demands) were less likely to participate when T2 data were collected. This suggests that our results are plausibly conservative, as we may have underestimated the increases in job demands and declines in job resources and well-being. Although we cannot check this given the lack of reference data, we do know that our study sample was similar to the population of healthcare professionals working in long-term care facilities in the Netherlands in terms of gender (88% and 91% female, respectively) and age (mean age of 49.7 and 43.2, respectively) [49, 50].

We performed multiple imputation with chained equations to deal with missing values and a robustness check, which yielded findings that were largely consistent with those of the main analysis. Only a few findings in the complete cases subsample showed small in differences with regard to statistical significance, with little differences in coefficient estimates’ direction and size.

### Implications for practice and policy

This longitudinal study provided new insight into how the working conditions and well-being of healthcare professionals in long-term care settings changed in the wake of surging infection rates during the COVID-19 pandemic. These professionals experienced increased workloads that resulted in poor work-related well-being, evidenced by increased work- and patient-related burnout as well as decreased work engagement. These findings suggest that policies targeting the maintenance of professionals’ well-being during pandemics should prioritize workload reduction. The achievement of this goal is very challenging, given the increase in long-term care staff shortages, but a solution is urgently required beyond the pandemic context and in preparation for potential future pandemics [37]. The recruitment and retention of more personnel in the long-term care workforce is a key priority. This macro-level problem can be addressed in part, for example, with the design of career paths involving promotion and/or opportunities for education and development at the local level [51, 52], but such strategies need to be accompanied by national strategies including the improvement of employment conditions (e.g., salaries, bonuses, and non-wage benefits such as childcare) and the public image of the profession [37, 51, 52].

Our findings also suggest that the promotion of collegial support can contribute to the prevention of work-related burnout and promotion of work engagement during a pandemic. It may, for example, be achieved by organizing team intervision (following emotional and traumatic experiences in care), peer-to-peer support, and/or informal team meetings [53]. Furthermore, decreased collegial support during hectic periods of the pandemic may be attributed to the lack of time for talking with colleagues. Thus, the addressing of root causes of time pressure (e.g., staff shortages) may be expected to foster collegial support during pandemics as well. Ultimately, the promotion of collegial support and reduction of workloads and/or other job demands facilitate professionals’ well-being and ability to provide high-quality care [12].

## Conclusions

This longitudinal study showed that healthcare professionals in long-term care settings in the Netherlands experienced increased workloads as COVID-19 infection rates surged between early and late 2021. This increase, and some professionals’ perceived decline in collegial support, resulted in increased burnout and decreased work engagement. These findings emphasize the urgency of reducing workloads and promoting collegial support to protect healthcare professionals’ work-related well-being in a pandemic context, especially during periods with high infection rates.

## Supplementary Information

**Additional file 1: Table SA.** Changes in job demands and job resources; complete cases subsample (*n* = 129). **Table SB.** Results of fixed-effects regression analyses predicting work-related well-being changes; complete cases subsample.

## Data Availability

The datasets used and/or analyzed during the current study are available from the corresponding author on reasonable request.
