# Supplementary material for: Changes in work conditions and well-being among healthcare professionals in long-term care settings in the Netherlands during the COVID-19 pandemic: a longitudinal study
Source: Hum Resour Health. 2023 Jul 28;21:59. doi: 10.1186/s12960-023-00847-z (PMC10385940; doi:10.1186/s12960-023-00847-z)
Supplement: Supplementary file 1 — Additional file 1: Table SA. Changes in job demands and job resources; complete cases subsample (n = 129). Table SB. Results of fixed-effects regression analyses predicting work-related well-being changes; complete cases subsample. [file 12960_2023_847_MOESM1_ESM.docx]

Table SA. Changes in job demands and job resources; complete cases subsample (n=129).

|  | T1 (Apr.-May 2021) | |  | T2 (Nov.-Dec. 2021) | |  | T2 versus T1 | |
| --- | --- | --- | --- | --- | --- | --- | --- | --- |
|  | Mean | (SD) |  | Mean | (SD) |  | ΔMean | (SE) |
| Job demands: |  |  |  |  |  |  |  |  |
| Workload | 3.374 | (0.718) |  | 3.523 | (0.803) |  | 0.149** | (0.048) |
| Emotional demands | 3.116 | (0.627) |  | 3.114 | (0.598) |  | -0.002 | (0.045) |
| Administrative burden | 3.121 | (0.795) |  | 3.231 | (0.759) |  | 0.110* | (0.047) |
|  |  |  |  |  |  |  |  |  |
| Job resources: |  |  |  |  |  |  |  |  |
| Supervisor support | 3.434 | (0.796) |  | 3.358 | (0.784) |  | -0.076 | (0.060) |
| Collegial support | 3.995 | (0.693) |  | 3.873 | (0.655) |  | -0.121* | (0.053) |
| Autonomy | 3.739 | (0.632) |  | 3.724 | (0.588) |  | -0.016 | (0.049) |

Notes: SD: Standard deviation; SE: Standard error; * *p* < .05, ** *p* < .01.

Table SB. Results of fixed-effects regression analyses predicting work-related wellbeing changes; complete cases subsample.

|  | Work-related burnout | |  | Patient-related burnout | |  | Work engagement | |
| --- | --- | --- | --- | --- | --- | --- | --- | --- |
|  | B | (SE) |  | B | (SE) |  | B | (SE) |
| Job demands: |  |  |  |  |  |  |  |  |
| Workload | 0.221** | (0.083) |  | 0.180† | (0.095) |  | -0.319* | (0.143) |
| Emotional demands | 0.276*** | (0.071) |  | 0.169* | (0.079) |  | 0.081 | (0.124) |
| Administrative burden | 0.239** | (0.074) |  | 0.189† | (0.101) |  | 0.075 | (0.131) |
|  |  |  |  |  |  |  |  |  |
| Job resources: |  |  |  |  |  |  |  |  |
| Supervisor support | -0.051 | (0.055) |  | -0.020 | (0.060) |  | 0.051 | (0.112) |
| Collegial support | -0.123* | (0.055) |  | -0.074 | (0.061) |  | 0.274* | (0.111) |
| Autonomy | -0.104† | (0.061) |  | -0.033 | (0.069) |  | 0.263* | (0.118) |
|  |  |  |  |  |  |  |  |  |
| Time period: |  |  |  |  |  |  |  |  |
| T1 (Feb. -May 2021) | Ref. |  |  | Ref. |  |  | Ref. |  |
| T2 (Nov.-Dec. 2021) | 0.002 | (0.034) |  | 0.011 | (0.042) |  | -0.092 | (0.068) |
|  |  |  |  |  |  |  |  |  |
| Number of observations | 258 |  |  | 258 |  |  | 258 |  |
| Number of persons | 129 |  |  | 129 |  |  | 129 |  |

Notes: All models estimated with robust standard errors; B: Coefficient estimate; SE: Standard error;
† p < .1, * p < .05, ** p < .01, *** p < .001.
